# Temperature Dependence of Hydrotropy

**DOI:** 10.1021/acs.jpcb.4c04619

**Published:** 2024-10-28

**Authors:** Seishi Shimizu, Nobuyuki Matubayasi

**Affiliations:** † York Structural Biology Laboratory, Department of Chemistry, University of York, Heslington, York YO10 5DD, U.K.; ‡ Division of Chemical Engineering, Graduate School of Engineering Science, Osaka University, Toyonaka, Osaka 560-8531, Japan

## Abstract

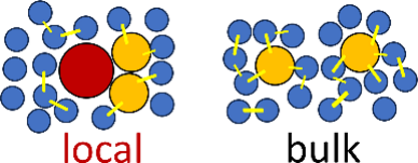

The solubility of hydrophobic solutes increases dramatically with the temperature when hydrotropes are added to water. In this paper, the mechanism of this well-known observation will be explained via statistical thermodynamics through (i) enhanced enthalpy-hydrotrope number correlation locally (around the solute) that promotes the temperature dependence and (ii) hydrotrope self-association in the bulk solution that suppresses the temperature dependence. The contribution from (i), demonstrated to be dominant for urea as a hydrotrope, signifies the weakening of interaction energies around the solute (local) than in the bulk that accompanies incoming hydrotrope molecules. Thus, studying hydrotropic solubilization along the temperature and hydrotrope concentration provides complementary information on the local-bulk difference: the local accumulation of hydrotropes around the solute, driven by the enhanced local hydrotrope self-association, is also accompanied by the overall local weakening of energetic interactions, reflecting the fluctuational nature of hydrotrope association and the mediating role of water molecules.

## 1 Introduction

**Figure 1:**
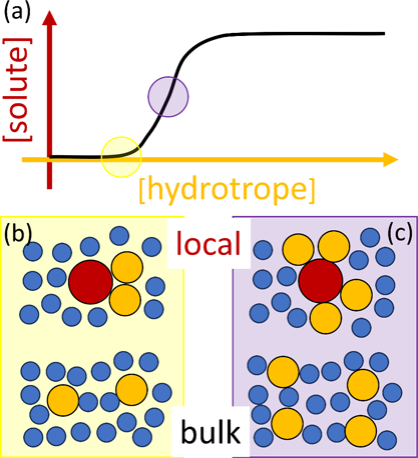
A schematic representation of hydrotropy according to the statistical thermodynamic fluctuation theory. (a) A typical solubility isotherm (i.e., the hydrotrope concentration dependence of solute solubility) identifying the two important regions in yellow (commonly referred to as the “minimum hydrotrope concentration”, with its mechanism illustrated in (b)) and purple (in (c)). (b) Around the minimum hydrotrope concentration, the hydrotrope-hydrotrope association is enhanced around the solute (local, compared to the bulk), driving the onset of solubilization (cf. the yellow region in (a)), which is a schematic summary of insights from the statistical thermodynamic fluctuation theory.^10−12^ The solute, water, and hydrotrope molecules are color-coded with red, blue, and orange, respectively, schematically representing stronger local hydrotrope association. (c) A steady increase in solubility is driven by the preferential interaction of hydrotropes around the solute; with the hydrotrope-hydrotrope association not as prominent as in (b), as has been represented schematically by comparable hydrotrope self-association between local and the bulk.

Hydrotropes have been employed in a range of industrial applications because of their significant ability to increase the solubility of hydrophobic solutes.^1−6^ However, their solubilization mechanism had long remained a mystery until a clarification from the statistical thermodynamic fluctuation theory (Figure 1):^7−10^ hydrotropic solubilization is driven by solute-hydrotrope association, which overcomes inefficiency caused by hydrotrope-hydrotrope association in the bulk phase.^7−10^ Moreover, the enhanced local hydrotrope self-association (around the solute) drives an onset of solubilization.^11−14^ However, all these mechanistic insights from statistical thermodynamics, supported also by ^1^H NMR.,^15^ were restricted to isothermal conditions.

**Figure 2:**
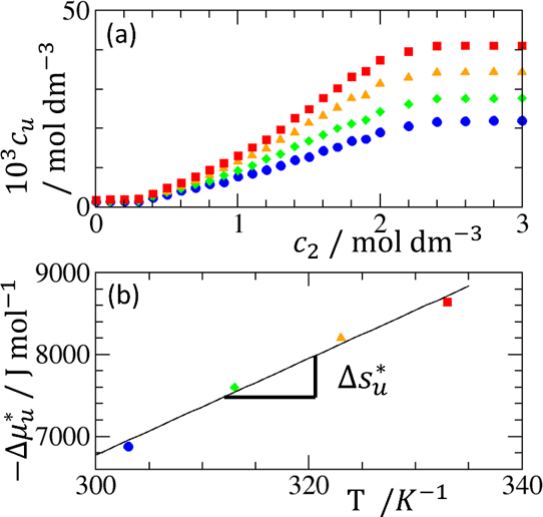
(a) Temperature-dependence of hydrotropy, illustrated by the solubilization of methyl benzoate (solute, abbreviated by MB) by urea (hydrotrope), at 303 K (blue squares), 313 K (green diamonds), 323 K (orange triangles), and 333 K (red squares), using the experimental data reported by Senthil et al.^54^ (b) Calculation of the entropy of transfer, Δ*s*_u_^*^, from pure water to saturated solubility (*c*_2_ = 2.8–3.0 M) from the gradient of −Δμ_u_^*^ = *RT* ln *c*_*u*_/*c*_*u*_^0^ (*c*_*u*_^0^: solubility in water, *c*_*u*_: solubility at saturation) against *T*, which yields a positive entropy of transfer, Δ*s*_u_^*^ = 58.9 J mol^–1^ K^–1^.

Solubility can usually be increased by raising the temperature.^16,17^ This “heat-solubilization” (as it will be referred to, with notable exceptions, e.g., small hydrophobic gases or salts) has been exploited routinely.^16,17^ Adding hydrotropes can further enhance heat solubilization (Figure 2a).^18−23^ Why, then, do hydrotropes enhance heat solubilization? According to the classical hypothesis, solubilization is caused by hydrotrope self-aggregation in the bulk aqueous solution.^2−4,24^ However, hydrotrope self-aggregation decreases with the temperature,^18,25,26^ while solubilization increases with the temperature^20,21^ (with a rare exception, as far as we know, of riboflavin solubility data in aqueous nicotinamide^18^), leading to a contradiction. Our statistical thermodynamic fluctuation theory, in its present form, is restricted to isothermal conditions, thereby incapable of explaining hydrotrope-intensification of heat-solubilization.^22,23^ Thus, how hydrotropes enhance heat-solubilization remains unexplained.

Our goal is twofold:

1. to explain how hydrotropes intensify heat solubilization; and
2. how (1) can shed light on the local-bulk difference of the solution structure.

To achieve this goal, we generalize our statistical thermodynamic fluctuation theory, restricted to isothermal conditions, to incorporate the temperature effects. Our specific objectives are

1. to identify the local-bulk difference of hydrotrope number-enthalpy correlation as the driving force for the intensification of heat-solubilization by hydrotropes;
2. to show that 1 signifies the local weakening of interaction energies caused by hydrotrope-solute association;
3. to clarify that hydrotrope-solute and hydrotrope-hydrotrope associations, the driving forces for solubilization, are nonstatic and water-mediated.

By achieving these objectives, we will be able to re-examine the traditional view on “water structure”, e.g., “urea as a structure breaker”,^27^ based chiefly on the entropy of transfer (Figure 2b). We will show how the classical view is related more intimately to the intensification of heat-solubilization, rather than to isothermal solubilization as has originally been intended.

## 2 Theory and Methods

### 2.1 Fluctuation Theory for Temperature-Dependent Solubility

Our goal is to clarify why heat-solubilization (i.e., solubility increase under raised temperatures) is promoted by hydrotropes added to the solution. To achieve this goal, we set up our system: a solvent mixture consisting of water (species 1) and hydrotrope (species 2), into which solute (species *u*) is dissolved in dilution. (In our cooperative solubilization theory, restriction to solutes in dilution was removed;^10^ this restriction was reinstated in this paper for mathematical simplicity necessitated by the temperature derivatives.) We adopt a partially open ensemble, {*T*, *P*, *N*_1_, μ_2_}, closed to water and open to hydrotrope molecules. (Note that *T*, *P*, *N*_*i*_, and μ_*i*_ denote the temperature, pressure, number, and chemical potential of species *i*, respectively.) According to the Gibbs phase rule,^28^ the degrees of freedom are *F* = 2 – 1 + 2 = 3. Keeping the pressure constant reduces the degrees of freedom to 2, leaving us two independent variables (i.e., *T* and hydrotrope concentration).

Our first objective is to formulate heat-solubilization and the influence of hydrotrope concentration thereupon. To this end, we start with the statistical thermodynamic expression for the solvation free energy of the solute,
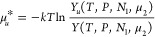
1where *k* is the Boltzmann constant and *Y*(*T*, *P*, *N*_1_, μ_2_) and *Y*_*u*_(*T*, *P*, *N*_1_, μ_2_) denote the partition functions of the partially open ensemble, {*T*, *P*, *N*_1_, μ_2_} and {*u*: *T*, *P*, *N*_1_, μ_2_}, respectively, where the latter refers to an inhomogeneous ensemble with a solute molecule fixed at the origin.^11^ To link μ_u_^*^ to solubilization, we introduce the solubilization ratio, *c*_*u*_/*c*_*u*_^*o*^, via the solubility in aqueous hydrotrope solution (*c*_*u*_) and that in pure water (*c*_*u*_^*o*^). The solubilization ratio is related to Δμ_u_^*^, i.e., the transfer free energy of a solute from pure water to aqueous hydrotrope solution (i.e., the difference in solvation free energy of a solute between hydrotrope solution and pure water), via
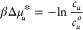
2where β = 1/*kT* has been introduced for shorthand. (In Appendix A, we have shown that the signatures of hydrotropy, summarized in Figure 1, manifest even when the logarithmic form of the isotherm () cannot be captured by the Setschenow equation.)

According to chemical thermodynamics, the temperature dependence of ln(*c*_*u*_/*c*_*u*_^o^) is central for comparing heat solubilization with and without the hydrotrope, via
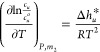
3awhere Δ*h*_u_^*^ is the enthalpy of transfer, defined as
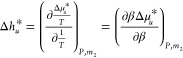
3bwhere the subscript *m*_2_ signifies “under constant hydrotrope molality”, via *m*_2_ = *N*_2_/(*N*_1_*M*_1_), where *M*_1_ is the molar mass of water. Note that Δ*h*_u_^*^ is a local thermodynamic quantity whose spatial contribution (from the microscopic solution structure) diminishes at a large distance from the fixed solute (see refs (29,30) in which the locality of thermodynamics has been defined and introduced, alongside the examples of nonlocal thermodynamic quantities). For this reason, we have employed isobaric ensembles throughout this paper which conforms to the experimental condition under which solubility measurements have been performed, instead of the grand canonical ensemble adopted as the generalization of the Kirkwood-Buff theory.^31−34^

However, in the {*T*, *P*, *N*_1_, μ_2_} and {*u*: *T*, *P*, *N*_1_, μ_2_} ensembles, hydrotrope fugacity, λ_2_ = *e*^βμ_2_^, rather than *m*_2_, is the natural measure of hydrotrope concentration. (Note that λ_2_ is related closely to hydrotrope activity, *a*_2_, hence to the molality *m*_2_, as will be clarified in Section 2.2.) Carrying out the β-derivative under constant λ_2_ yields
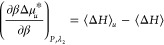
4where Δ*H* = *H* – *H*^o^ is the difference in enthalpy between hydrotrope solution and pure water (see Appendix B for derivation. Note that *H*^o^ is already an ensemble average in the system of pure water, hence is not subjected to the ensemble averaging operations in eq 4). Thus, under constant λ_2_, the local-bulk difference in average Δ*H* is the quantity responsible for the heat-solubilization difference between the presence and absence of hydrotropes. (In Section 2.2, ⟨Δ*H*⟩_*u*_ – ⟨Δ*H*⟩ and Δ*h*_u_^*^ are shown to be close in values.)

How effective a hydrotrope is in enhancing heat solubilization can be quantified by the gradient of ⟨Δ*H*⟩_*u*_ – ⟨Δ*H*⟩ with respect to hydrotrope concentration. Our goal is to evaluate this derivative while linking it to statistical thermodynamic quantities that convey a clear physical meaning. This goal can be achieved by taking λ_2_ as the variable. Differentiating eq 4 with respect to λ_2_ under constant β, we obtain (see Appendix B):
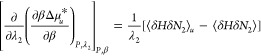
5Following the discussion in Appendix B, the λ_2_-derivative in eq 5 can be rewritten as the ln*a*_2_-derivative (where *a*_2_ is hydrotrope activity), as
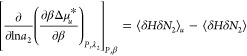
6Converting the variable from ln*a*_2_ to hydrotrope concentration is straightforward with the use of hydrotrope molality, *m*_2_, through a well-known result from the Kirkwood-Buff theory of binary mixtures (see eq 21 of ref (35)),
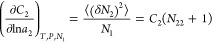
7awhere *C*_2_ = *N*_2_/*N*_1_ = *M*_1_*m*_2_,^13,36−39^ with *M*_1_ being the molecular weight of water. Note that eq 7a is applicable to bulk solutions without the solute, as well as in the presence of the solute under phase equilibrium at arbitrary concentration.^10^ In the following, we consider the solute at infinite dilution, for which eq 7a for the bulk solution will be employed. *N*_22_ is the excess number of hydrotropes around a probe hydrotrope, as the measure of bulk phase self-association, defined as
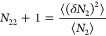
7bCombining eqs 6 and 7a yields
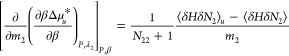
7cThus, eq 7c is the central result of this paper, from which the hydrotrope effect on heat-solubilization will be made clearer through its simplification in Section 2.2.

The simplicity in the form of eq 7c comes from the isobaric, partially open ensembles adopted for the local and bulk systems and the inhomogeneous solvation theory for the local system. Previously, local fluctuations have been formulated chiefly in the grand canonical (open isochoric) ensemble in extending the Kirkwood-Buff theory of number–number correlation to incorporate number-energy correlations.^31−34^ The advantage of partially open ensembles has been recognized for their ease in connecting experiments to theory,^39−43^ which is often cumbersome for the approaches founded on the grand canonical ensembles.^40^ In addition, the inhomogeneous solvation theory, in its ability to treat a fixed solute as an external field, is not only suitable for local thermodynamics but also for simplifying complex expressions involving ternary correlations (e.g., solute-hydrotrope-hydrotrope) to the conditional binary (e.g., hydrotrope-hydrotrope in the presence of the solute).^41−46^ For these reasons, we have adopted the partially open ensembles and inhomogeneous solvation theory for interpretive clarity, which will be demonstrated in Section 3.

### 2.2 Simplification for Experimental Data Analysis

Our goal is to simplify eq 7c to clarify how hydrotropes intensify heat-solubilization. To this end, we will rewrite eq 7c to be in better conformity with experimental practice.

#### 2.2.1 Equivalence of the Enthalpies of Transfer from the Fluctuation Theory and Chemical Thermodynamics

Here, we show that the two β-derivatives, eqs 3b (constant *m*_2_) and 4 (constant λ_2_), are close in value to one another, such that
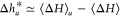
8aso that the enthalpy of transfer, Δ*h*_u_^*^, commonly used in solvation thermodynamics,^47^ can be adopted for the fluctuation theory. To this end, the following relationship, derived in Appendix C, will play a key role:
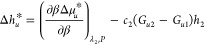
8bwhere *c*_2_ is the molarity of the hydrotrope, *h*_2_ is the partial molar enthalpy of the hydrotrope, and *G*_*ui*_ is the Kirkwood-Buff integral between solute (*u*) and species *i*. (We emphasize that eq 8b, while involving the Kirkwood-Buff integrals defined in the grand canonical ensemble, is connected to the enthalpy of solvation in the isobaric ensemble.) The order-of-magnitude analysis of *c*_2_(*G*_*u*2_ – *G*_*u*1_)*h*_2_ in eq 8b, carried out in Appendix C, shows that this term makes a minor contribution to urea as a hydrotrope. Consequently, eq 6 can be simplified as
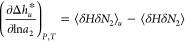
9Note, in eq 9, that constant *T* is equivalent to constant β. We emphasize that eq 9 is an approximation specific for urea as a hydrotrope, subject to the negligibility of *c*_2_(*G*_*u*2_ – *G*_*u*1_)*h*_2_ in eq 8b. If this term is not negligible for a hydrotrope-solute combination, eq 8b must be used to evaluate , required for our general result (eq 7c), from Δ*h*_u_^*^ and *c*_2_(*G*_*u*2_ – *G*_*u*1_)*h*_2_, both of which can be evaluated when a full set of experimental data is available (Appendix C).

#### 2.2.2 Calculating Enthalpy of Transfer from Solubility Data

**Figure 3:**
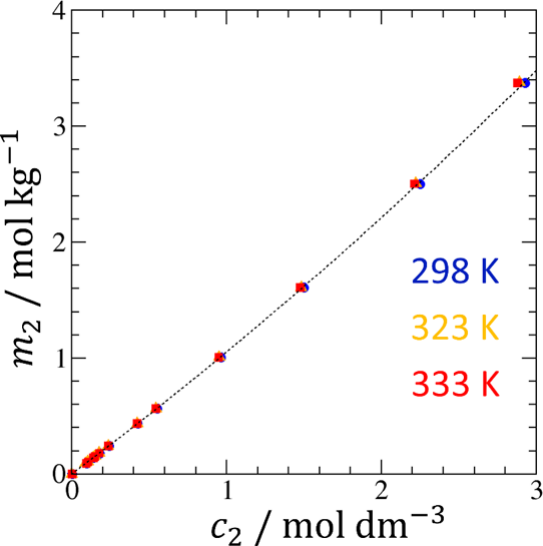
Molarity-molality relationship, based on the experimental density data of aqueous urea solutions at 298 K (blue circles), 323 K (orange triangles), and 333 K (red squares) at 1 atm published by Makarov and Egorov,^49^ which shows little temperature dependence. The dotted line is the fitting equation at 303 K (based on the density data at 298 and 303 K), *m*_2_ = 0.996*c*_2_ + 0.0544*c*_2_^2^, used throughout this work.

**Figure 4:**
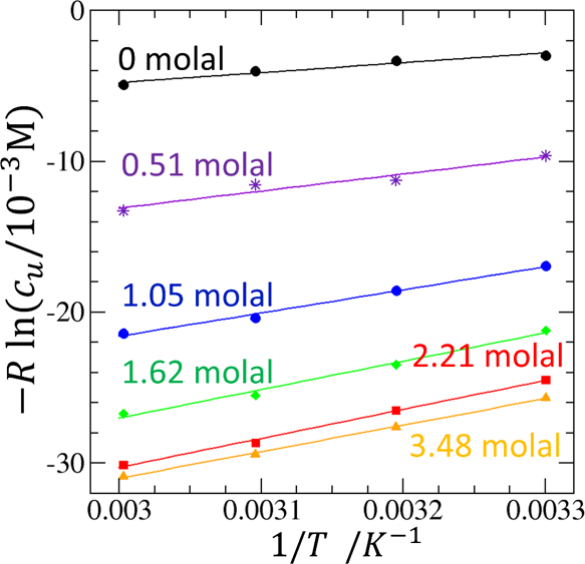
1/*T*-dependence of −*R* ln *c*_*u*_ (*c*_*u*_: the solubilities of MB in aqueous urea solutions) for the calculation of the enthalpy of transfer, Δ*h*_u_^*^, via eq 10. Shown here are the sample data fitting at 0 molal (black circles), 0.51 molal (purple asterisks), 1.05 molal (blue circles), 1.62 molal (green diamonds), 2.21 molal (red squares), and 3.48 molal (orange triangles), with the corresponding linear regression. The calculated Δ*h*_u_^*^ will be presented in Figures 5 and 6.

Calculating the enthalpy of transfer, Δ*h*_u_^*^, from solubility data via eq 3 can be facilitated significantly by establishing
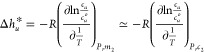
10Since the molality *m*_2_, by definition, is independent of the temperature, establishing eq 10 is equivalent to the negligible temperature-dependence of molarity *c*_2_ for aqueous hydrotrope solutions within the temperature range covered by the solubility data. Note that eq 10 is valid for solutes with low solubility for which *c*_2_ is not affected by *c*_*u*_, i.e., the presence of the solute in the solution. This paper focuses on urea as a hydrotrope, which offers a rare combination of available density and activity data^48,49^ covering the entire range of concentrations and temperature range for the solubility data,^19,50−54^ in contrast to the severe limitations of data availability for other hydrotropes. As shown in Figure 3, the molarity-molality relationship shows no temperature variation between 298 to 333 K, which justifies eq 10. (This justification is underscored by another route via thermal expansion as demonstrated in Appendix D.) Thus, Δ*h*_u_^*^ can be evaluated via eq 10 directly from the experimental solubility data reported at a regular *c*_2_ interval, as demonstrated by Figure 4 for the solubility data of methyl benzoate (MB).^54^

In addition to MB, we have used the published solubility data of p-Aminobenzoic Acid (AB),^50^ butyl acetate (BA),^19^ benzyl benzoate (BB),^51^ butyl stearate (BS),^52^ and ethylbenzene (EB),^53^ all at 303, 313, 323 and 333 K between 0 and 3 M urea as the hydrotrope, whose results will be presented in Section 3.

## 3 Results and Discussion

### 3.1 Hydrotrope Effect on Heat-Solubilization

Our goal is to clarify why hydrotropes promote heat-solubilization. To this end, we begin by summarizing our achievements in Section 2. First, the enthalpy of transfer, Δ*h*_u_^*^, characterizes the heat-solubilization difference between hydrotrope solution and pure water, which can be obtained via eq 10, namely the temperature dependence of , in which the constant molality condition can be approximated by constant molarity (see eq 10). According to eq 3a, a positive Δ*h*_u_^*^ is responsible for enhanced heat-solubilization in the presence of hydrotropes. Second, how heat-solubilization changes with increasing hydrotrope concentration can be captured by,
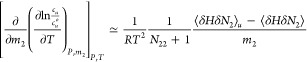
11awhich can be derived by combining eqs 3a, 7c, and 10. On the left-hand side of eq 11a, the symmetry of partial differentiation yields
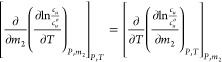
11bwhich states the equivalence between “the hydrotrope effect on heat-solubilization” (the left-hand side) and “the temperature effect on hydrotrope-solubilization” (the right-hand side).

According to eq 11a, our central result, the enhancement of heat solubilization by hydrotropes is caused by the competition between the two contributions. The first contribution is ⟨δ*H*δ*N*_2_⟩_*u*_ – ⟨δ*H*δ*N*_2_⟩ (normalized by *m*_2_, the hydrotrope molality) drives up ; a positive (⟨δ*H*δ*N*_2_⟩_*u*_ – ⟨δ*H*δ*N*_2_⟩)/*m*_2_ signifies the increased correlation between the number of hydrotropes and the enthalpy when the solute is present. The second contribution is the hydrotrope self-association in the bulk, quantified by *N*_22_ (i.e., the excess number of hydrotrope around a probe hydrotrope), which lowers heat-solubilization, acting similarly to how hydrotrope self-association in the bulk decreases the solubilization efficiency of the hydrotrope.^7,8^

**Figure 5:**
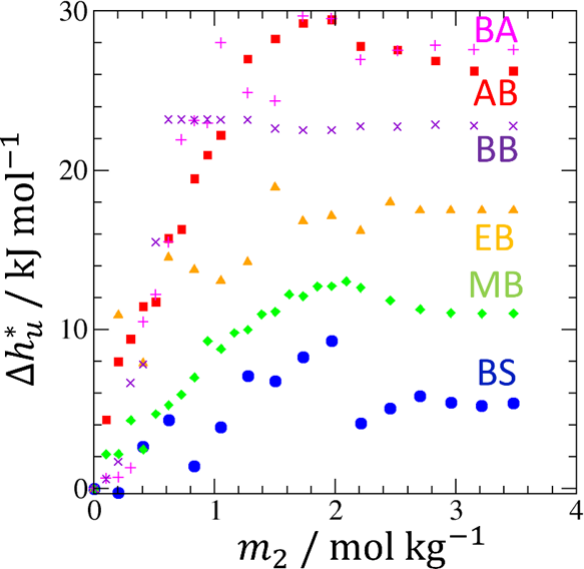
Hydrotrope concentration (*m*_2_: molality) dependence of Δ*h*_u_^*^, the enthalpy of transfer from pure water to aqueous urea solution for the solutes BA (magenta crosses), AB (red squares), BB (violet xs), EB (orange triangles), MB (green diamonds), and BS (blue circles).

With this preparation, here we analyze experimental data. First, we show how Δ*h*_u_^*^ changes with hydrotrope concentration based on experimental solubility data (Figure 5). According to the procedures outlined in Section 2.2, we observe the increase of Δ*h*_u_^*^ with *m*_2_ at lower *m*_2_ (generally corresponds to the first half of sigmoidal solubility curves) and saturation of Δ*h*_u_^*^ at higher *m*_2_ (in Figure 5). Thus, we obtain a positive  at lower *m*_2_ and  at higher *m*_2_. The gradient, , can be attributed solely to (⟨δ*H*δ*N*_2_⟩_*u*_ – ⟨δ*H*δ*N*_2_⟩)/*m*_2_ in eq 11a, because *N*_22_ + 1 ≃ 1 for urea, due to its well-known property of forming a near-ideal mixture with water.^7,8^

At lower urea concentration, a positive (⟨δ*H*δ*N*_2_⟩_*u*_ – ⟨δ*H*δ*N*_2_⟩)/*m*_2_ signifies a stronger correlation around the solute (⟨δ*H*δ*N*_2_⟩_*u*_) between hydrotrope accumulation (δ*N*_2_ > 0) and weakened interaction (δ*H* > 0) than in the bulk (⟨δ*H*δ*N*_2_⟩). Note that *H* is the sum of all the interaction energies, not only between hydrotrope molecules but also between hydrotrope and water as well as water and water. Thus, the enhancement of heat-solubilization by hydrotropes has been attributed to the weakening of interaction energy by hydrotropes coming into the solute’s locality more prominently so than in the bulk. We emphasize that the weakening of interaction energy is not contradictory to the accumulation of hydrotropes around the solute and the enhancement of hydrotrope-hydrotrope association (Figure 1b), both of which are essentially the potential of mean force interactions. In Section 3.2, we will synthesize the insights from enthalpy-number and number–number correlations to clarify the nature of hydrotrope associations.

For hydrotropes with stronger bulk-phase self-aggregation, *N*_22_ + 1 is larger than 1.^7,8^ Since *N*_22_ + 1 is in the denominator of eq 11a, it contributes to attenuate the hydrotrope effect on heat solubilization. Thus, the self-association of hydrotrope in the bulk phase counteracts not only isothermal solubilization (which is driven by preferential solute-hydrotrope interaction)^7,8^ but also the enhancement of heat solubilization (which is driven by the local strengthening of number-enthalpy correlation).

### 3.2 Comparison to the Classical View

The accumulated hydrotropes around the solute, responsible for solubilization, also weaken the interaction energy in the locality of the solute, as shown in Section 3.1. Such a role of urea in the vicinity of the solute is analogous to “urea as a structure breaker”^27^ from the classical view, i.e., how urea weakens the hydrophobic effect by breaking the hydrogen bond network of water responsible for the hydrophobic effect. Such a view, however, has also been subjected to questioning from spectroscopy,^55^ from the mixing ideality of urea and water,^56^ as well as from direct simulations of the distribution functions of water and urea molecules and their statistical thermodynamic link to the solvation free energies.^57,58^ Note that the classical view, despite its aim to elucidate the solvation free energy, was unwittingly referring to the role of urea on the temperature dependence of solubility because of its focus on the (delicate) balance of entropy and enthalpy, rather than the clearly observed dependence of the enthalpy on the hydrotrope concentration.

#### 3.2.1 Classical View

**Figure 6:**
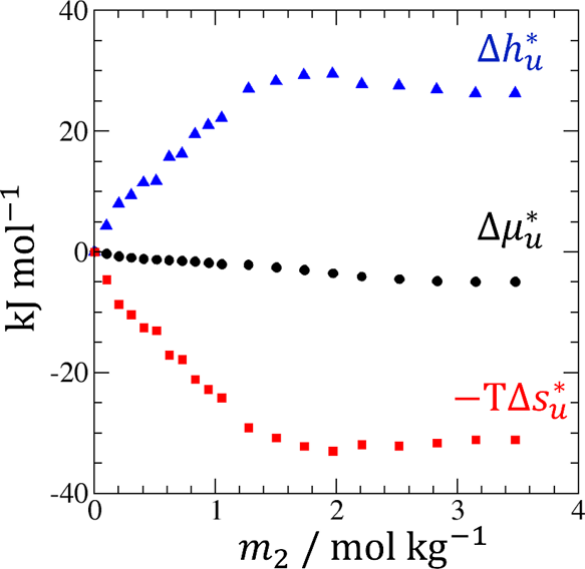
Transfer free energy Δμ_u_^*^ (black circles), directly related to solubilization via Δμ_u_^*^ = −*RT* ln (*c*_*u*_/*c*_*u*_^o^), is a small difference between the compensating transfer enthalpy Δ*h*_u_^*^ (blue triangles) and entropy −*T*Δ*s*_u_^*^ (red squares). Δμ_u_^*^ of AB in aqueous urea solutions were calculated from the solubility data reported by Senthil et al. at 303 K.^54^ Δ*h*_u_^*^ was calculated in the method described in Figure 4.

Using simplified models, the classical view aimed to understand the origin of solubilization (i.e., Δμ_u_^*^ = Δ*h*_u_^*^ – *T*Δ*s*_u_^*^ < 0) based on its entropic contribution −*T*Δ*s*_u_^*^.^27,59^ We emphasize that Δ *h*ere refers to “aqueous solubilizer solution minus pure water”. Structure breaking leads to Δ*s*_u_^*^ > 0 (i.e., a more positive solvation entropy in aqueous solubilizer solution than in water), hence to a negative −*T*Δ*s*_u_^*^,^27^ which is consistent with Figure 6. However, the difficulty of this approach is well-known: solubilization (Δμ_u_^*^ < 0) is a small difference between the two large contributions, Δ*h*_u_^*^ and *T*Δ*s*_u_^*^, as shown in Figure 6. Indeed, structure breaking also leads to a positive Δ*h*_u_^*^ through weakened intermolecular interaction energies, which is a well-known phenomenon, referred to as the entropy-enthalpy compensation.^59,60^ Thus, the classical view of solubilization remains speculative unless we understand why the contribution from −*T*Δ*s*_u_^*^ is (slightly) greater than Δ*h*_u_^*^. Such an approach remains challenging because Δ*s*_u_^*^ involves complex expressions arising from multiple-body intermolecular correlations even in the absence of the hydrotropes.^30,61,62^

#### 3.2.2 Fluctuation Theory

Unlike the classical view, our fluctuation theory is founded on the exact, model-free relationships derived from the principles of statistical thermodynamics. First, *isothermal* solubilization by adding hydrotropes (i.e., the decreasing Δμ_u_^*^ with hydrotrope concentration, ) was attributed to preferential solute-hydrotrope interaction,^7−9^ whose onset around the minimum hydrotrope concentration originates from the enhanced hydrotrope-hydrotrope association around the solute.^10−12^ Such a conclusion is free from the conundrum encountered by splitting Δμ_u_^*^ into Δ*h*_u_^*^ and *T*Δ*s*_u_^*^ as adopted by the classical view. Second, the structure-breaking (i.e., weakened local interaction energies or the number-enthalpy correlation) by hydrotrope was linked to the competition between ⟨δ*H*δ*N*_2_⟩_*u*_ – ⟨δ*H*δ*N*_2_⟩ and *N*_22_, which drives the signature of the hydrotrope effect on heat-solubilization (see eq 11a). This conclusion is different from the speculative link between “structure breaking” and Δμ_u_^*^ according to the classical view and would be a way to define “structure breaking” in a manner directly relatable to the thermodynamics of solubilization.

Thus, the fluctuation theory has shown that the local structure breaking by hydrotropes is the cause of their ability to enhance heat-solubilization.

### 3.3 Local versus Bulk Behavior of Hydrotropes

We have identified the three signatures of hydrotropy and their respective microscopic mechanisms. The signatures are

**Figure 7:**
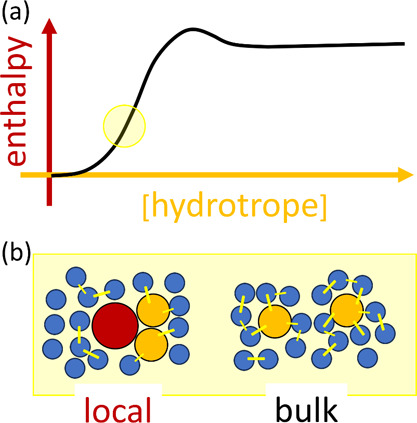
A schematic representation of local hydrotrope association. (a) A typical hydrotrope concentration dependence of the enthalpy of transfer, Δ*h*_u_^*^, in which the important region is highlighted in yellow. (b) The local hydrotrope-solute and hydrotrope-hydrotrope association around the solute (left) is accompanied by more unfavorable energetic interactions than in the bulk (right), as represented schematically by the reduced number and strength of intermolecular bonds (represented by yellow lines with different lengths and thickness) that contribute to lower the interaction energy.

A. Isothermal solubilization by hydrotropes (Figure 1a);^7−9^
B. The isothermal onset of solubilization by hydrotropes at the minimum hydrotrope concentration (Figure 1a, yellow highlighted region);^10−12^ and
C. Enhancement of heat-solubilization by hydrotropes (Figures 2 and 7a).

The mechanisms underlying A–C are

1. The dominance of solute-hydrotrope association over bulk hydrotrope-hydrotrope association (Figure 1b);^7−9^
2. Enhancement of hydrotrope association locally than in the bulk (Figure 1b);^10−12^ and
3. Interaction energy being weaker locally than in the bulk (Figure 7b).

Since they refer to different thermodynamic functions and conditions, their insights are complementary to one another.

Here, we show that synthesizing A–C will lead to a clearer understanding of hydrotropy on a microscopic scale: local (=around the solute) hydrotrope accumulation is accompanied also by a local weakening of overall interaction energy (i.e., local structure breaking). Such a statement would be counterintuitive if “interaction” simply meant the formation of the dimers, trimers, and clusters of hydrotropes. Indeed, “structure breaking” is accompanied by solute-hydrotrope and local hydrotrope-hydrotrope associations (1–3), as shown schematically in Figure 7b. However, 1–3 can be rationalized by understanding hydrotrope-solute and hydrotrope-hydrotrope interactions as the potentials of mean force (Figure 7b), that, by definition, are mediated by water.^63^ In this framework, enhanced self-association of hydrotrope around the solute is not contradictory to the weakening of energetic interactions around the solute, as depicted schematically by Figure 7b, since the former reflects the free energy that includes the energetic and entropic contributions. We emphasize that the potential of mean force is founded on the molecular distribution function^63^ that can capture the statistical distribution of fluctuating, nonstatic hydrotrope molecules around the solute, instead of hypothesizing hydrotrope clusters with well-defined stoichiometry.

Thus, synthesizing hydrotropy along concentration and temperature axes has led to an elucidation that hydrotrope accumulation and local self-association enhancement are far from static and stoichiometric. Such a fluctuating nature of the hydrotrope around the solute is responsible for the strong temperature dependence of hydrotropic solubilization. (Note that “fluctuation” here is thermodynamic, arising from the existence of broad ensembles of structures in the ensemble, rather than kinetic fluctuations in time.)

## 4 Conclusions

Hydrotropes often enhance the heat-solubilization of solutes.^18−23^ To elucidate the origin of this well-known and well-exploited yet hitherto unexplained experimental observation, we extended our statistical thermodynamic fluctuation theory of hydrotropy^7−12^ to incorporate the temperature effects (by taking advantage of the formal simplicity afforded by the inhomogeneous solvation theory^41−46^ and partially open ensembles^39−43^ as an alternative approach to the extension of the Kirkwood-Buff theory.^31−34^). The hydrotrope enhancement of heat-solubilization is strengthened by (i) enhanced hydrotrope number-enthalpy correlation around the solute and weakened by (ii) hydrotrope self-association in the bulk solution.

The enhanced hydrotrope number-enthalpy correlation around the solute can be interpreted as the local “structure breaking”. This novel insight, based on a rigorous statistical thermodynamic theory, is different from the classical speculations about a link between “structure breaking” and solubilization.^27^ Instead, local structure breaking by hydrotropes intensifies heat-solubilization, as has been clarified by the statistical thermodynamic fluctuation theory (eq 11a).

Thus, studying solubilization along the hydrotrope concentration and the temperature axes leads to a clarification of the local behavior of hydrotropes. Both the hydrotrope-solute association (as the driving force for solubilization)^7−9^ and the locally enhanced hydrotrope-hydrotrope association (cause for the onset of solubilization at the minimum hydrotrope concentration)^10−12^ weaken the local interaction energy, as shown by the local-bulk difference in number-enthalpy correlation. This clarifies the fluctuating, nonstatic, and water-mediated nature of local hydrotrope associations, which are responsible for the enhanced heat-solubilization by hydrotropes.

## Figures and Tables

**Figure 8:**
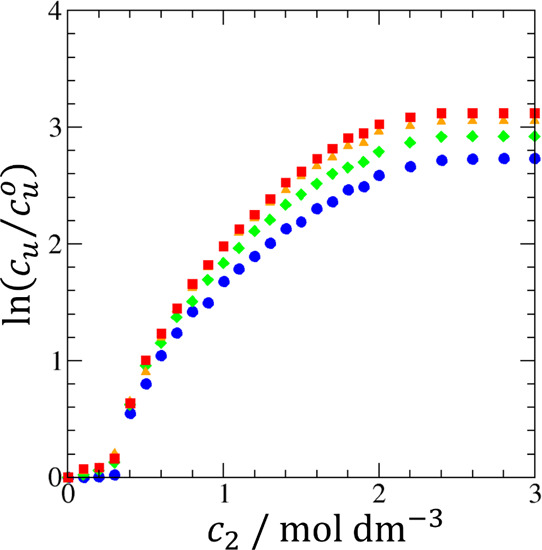
Replotting the solubility isotherm of MB in aqueous urea solutions (Figure 2a) as the logarithmic solubilization (ln(*c*_*u*_/*c*_*u*_^o^)) of MB against urea concentration (*c*_2_), which exhibits a nonlinear behavior that cannot be captured by the Setschenow equation (eq A1) and preserves the signatures of hydrotropy summarized in Figure 1a (i.e., the minimum hydrotrope concentration and saturation).

**Figure 9:**
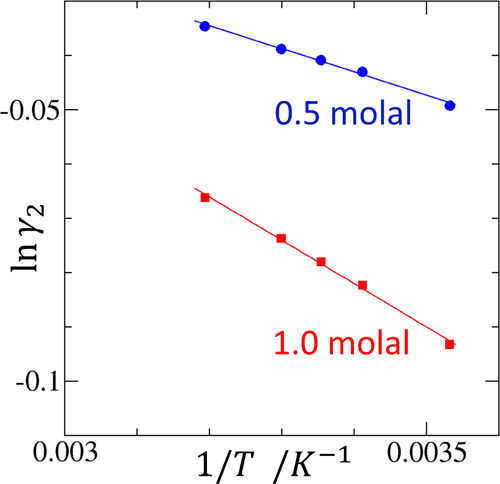
Partial molar enthalpy of urea, *h*_2_, can be calculated from the 1/*T*-dependence of lnγ_2_ (where γ_2_ is in the molal concentration basis). The experimental data for 0.5 and 1.0 molal urea solutions (taken from Stokes^48^), in blue circles and red squares, have been fitted with lnγ_2_ = −42.7*T*^–1^ + 0.102 (blue solid line) and lnγ_2_ = −79.9*T*^–1^ + 0.190 (red solid line), yielding (via eq C4 in Appendix C) *h*_2_ = −0.35 and −0.66 kJ mol^–1^ for 0.5 and 1.0 molal, respectively.

## Appendix A: A Non-Setschenow Behavior of Hydrotropy

The signature of hydrotropy, i.e., the sigmoidal functional shape of the solubility isotherm with the “minimum hydrotrope concentration” and saturation, has commonly been described using the plot of solubility against hydrotrope concentration (Figure 1). In contrast, the Setschenow equation,

A1

has been commonly used to quantify solubility via the Setschenow constant, *k*_S_, based on the logarithmic plot of solubilization against the hydrotrope concentration. However, our example for hydrotropy, i.e., the solubilization of MB in aqueous urea solutions (Figure 2a), cannot be captured by the Setschenow constant; Figure 8 demonstrates a nonlinear dependence of  on *c*_2_, showing that the sudden onset of solubilization at the “minimum hydrotrope concentration” and saturating solubilization are not the artifacts of adopting *c*_*u*_ for solubility isotherms.

## Appendix B: Fluctuation Theory

First, we derive eq 4, starting from eq 1. To do so, we start with the expressions of the partially open partition functions, i.e.,

B1a

B1b

where *Q* and *Q*_*u*_ are the canonical partition functions and λ_2_, the fugacity of hydrotrope, is defined as λ_2_ = *e*^βμ_2_^. Carrying out the β-derivative of eq 1, we arrive at

B2

where ⟨*H*⟩_*u*_ and ⟨*H*⟩ are the enthalpies of the inhomogeneous and homogeneous systems, respectively, defined as

B3a

B3b

With the above preparation, now we derive eq 4. To do so, we note that eq B2 is valid for all hydrotrope concentrations. Consequently, subtracting the pure water version of eq B2 yields

B4

where *H*^o^ is the enthalpy in pure water. Introducing Δ*H* = *H* – *H*^o^ yields eq 4.

Second we derive eq 5. To do so, it is useful to note that

B5

Carrying out a μ_2_-differentiation of eq B3a, we obtain

B6

Evaluating the derivative of eq B3b similarly, while noting that *H*^o^ does not depend on μ_2_, we obtain

B7

which is eq 5.

Finally, we derive eq 6 from eq 5 through a variable conversion from λ_2_ to *a*_2_, by remembering the, λ_2_ = *e*^βμ_2_^. Taking advantage of the constant β in eq B7 (eq 5), such that

B8

Using β*d*μ_2_ = *d* ln *a*_2_ = *da*_2_/*a*_2_, we obtain

B9

which leads straightforwardly to eq 6.

## Appendix C: Relating Constant Fugacity to Constant Mole Ratio

Here, we demonstrate the accuracy of

C1

via an order-of-magnitude analysis using experimental data. In this Appendix, all the partial differentiations are done at constant pressure, and the pressure *P* as a fixed parameter will be omitted in the expressions for the partial derivatives. Noting that the constant λ_2_ is equivalent to constant βμ_2_ because of λ_2_ = *e*^βμ_2_^, we start with the following change of variables

C2a

which can be simplified (under constant β, which is equivalent to constant *T*) as

C2b

Using the following well-known results from the Kirkwood-Buff theory^24^ and chemical thermodynamics,

C2c

where *h*_2_ is the partial molar enthalpy of the hydrotrope. Combining eqs C2b and C2c yields

C3a

which, via eqs 2 and 3b (and noting, again, that constant λ_2_ is equivalent to constant βμ_2_), can be rewritten as

C3b

To establish the accuracy of eq C1, we need to demonstrate that *c*_2_(*G*_*u*2_ – *G*_*u*1_)*h*_2_ in eq C3b makes a minor contribution compared to other terms. Since *c*_2_(*G*_*u*2_ – *G*_*u*1_) has been reported for aqueous hydrotrope solutions, here we focus on the remaining factor, *h*_2_. Using the molality-based activity coefficient, γ_2_, *h*_2_ can be evaluated via

C4

With the above preparation, now we carry out an order-of-magnitude analysis on *c*_2_(*G*_*u*2_ – *G*_*u*1_)*h*_2_ in eq C3b, by taking urea as an example hydrotrope, because of its common use as a hydrotrope for which all the physical properties data necessary for our analysis is available. First, at the peak of *G*_*u*2_ – *G*_*u*1_ with respect to its *c*_2_ dependence, most sharply for ethylbenzene (EB)^8^ with *G*_*u*2_ – *G*_*u*1_ ≃ 2.5 dm^3^ mol^–1^ at *c*_2_ ≃ 0.6 mol dm^–3^, *c*_2_(*G*_*u*2_ – *G*_*u*1_) ≃ 1.5. Second, *h*_2_, evaluated via the temperature-dependence of the molality-based activity coefficient of urea, γ_2_, at the urea concentration of 0.5 molal (close to the *c*_2_(*G*_*u*2_ – *G*_*u*1_) peak) between 10 and 40 °C yields *h*_2_ = −0.35 kJ mol^–1^ (Figure 9). Combining all above leads to an estimation of *c*_2_(*G*_*u*2_ – *G*_*u*1_)*h*_2_ ≃ −0.53 kJ mol^–1^, which is minor compared to Δ*h*_*u*_^*^ ∼ 10 kJ mol^–1^ (see Figure 5) in the same hydrotrope concentration region. This approximation is still valid at higher hydrotrope concentrations where (*G*_*u*2_ – *G*_*u*1_) significantly decreases in magnitude while Δ*h*_*u*_^*^ increases to ∼18 kJ mol^–1^. Thus, we have justified eq C1.

We emphasize that the limited availability of thermodynamic data has restricted our analysis to choose urea as the sole example of hydrotrope in this paper. When the approximation (eq C1) breaks down, required for the fluctuation theory can be calculated via eq C3b, through a direct evaluation of (i) Δ*h*_*u*_^*^ from the temperature-dependent solubility data (eq 3a), (ii) *h*_2_ from the temperature-dependent activity data (eq C4), and (iii) *c*_2_(*G*_*u*2_ – *G*_*u*1_) from the Kirkwood-Buff theory based on a combination of the solubility and activity data.^64^

## Appendix D: Use of Hydrotrope Molarity in Data Analysis

Here, we show that the effect of thermal expansion on hydrotrope is quite negligible, hence the constant molality condition, required by chemical thermodynamics, can be approximated by constant hydrotrope molarity. When the temperature rises by Δ*T*, the relative change of molarity, induced by the thermal expansion of the solution, can be expressed as

D1

where α_V_ is the thermal expansion coefficient of the aqueous hydrotrope solution. Here, we carry out the order-of-magnitude estimation, using α_V_ by that of pure water. At 318 K (which is right in the middle of the temperature range, 303–333 K, of the solubility data by Nagendra Gandhi and co-workers), α_V_ = 4.22 × 10^–4^ K^–1^ as reported by Kell.^65^ (Note that α_V_ of aqueous urea solutions are also in the order of α_V_ ∼ 10^–4^ K^–1^.) Taking the temperature interval Δ*T* = 30 K, the relative change of molarity is estimated as

D2

Thus, the constant *m*_2_ (hydrotrope molality) condition, required by chemical thermodynamics, can be approximated by a constant *c*_2_ condition.
